# The transcriptome of human mammary epithelial cells infected with the HCMV-DB strain displays oncogenic traits

**DOI:** 10.1038/s41598-018-30109-1

**Published:** 2018-08-22

**Authors:** Fatima Al Moussawi, Amit Kumar, Sébastien Pasquereau, Manoj K. Tripathy, Walid Karam, Mona Diab-Assaf, Georges Herbein

**Affiliations:** 1 Department Pathogens & Inflammation-EPILAB, UPRES EA4266, University of Franche-Comté, University of Bourgogne Franche-Comté, F-25030 Besançon, France; 2 Université Libanaise, Beyrouth, Lebanon; 3 Department of Virology, CHRU Besancon, F-25030 Besançon, France

## Abstract

Increasing evidence indicates that human cytomegalovirus (HCMV) populations under the influence of host environment, can either be stable or rapidly differentiating, leading to tissue compartment colonization. We isolated previously from a 30-years old pregnant woman, a clinical isolate of HCMV, that we refered to as the HCMV-DB strain (accession number KT959235). The HCMV-DB clinical isolate demonstrated its ability to infect primary macrophages and to upregulate the proto-oncogene Bcl-3. We observed in this study that the genome of HCMV-DB strain is close to the genomes of other primary clinical isolates including the Toledo and the JP strains with the later having been isolated from a glandular tissue, the prostate. Using a phylogenetic analysis to compare the genes involved in virus entry, we observed that the HCMV-DB strain is close to the HCMV strain Merlin, the prototype HCMV strain. HCMV-DB infects human mammary epithelial cells (HMECs) which in turn display a ER−/PR−/HER2− phenotype, commonly refered to as triple negative. The transcriptome of HCMV-DB-infected HMECs presents the characteristics of a pro-oncogenic cellular environment with upregulated expression of numerous oncogenes, enhanced activation of pro-survival genes, and upregulated markers of cell proliferation, stemcellness and epithelial mesenchymal transition (EMT) that was confirmed by enhanced cellular proliferation and tumorsphere formation *in vitro*. Taken together our data indicate that some clinical isolates could be well adapted to the mammary tissue environment, as it is the case for the HCMV-DB strain. This could influence the viral fitness, ultimately leading to breast cancer development.

## Introduction

Breast cancer, the most common cancer diagnosed among women, exhibits heterogeneous molecular characteristics. Several types of breast cancer have been identified based on the differential gene expression patterns. These groups include among others the normal breast epithelial-like, the luminal epithelial type A and type B, the basal-like and the claudin low groups^1^. Genetic risk factors and environmental risk factors are some of the etiologic factors involved in breast cancer^2^. Of all worldwide cancers close to one-fifth could involve infectious agents including viruses, as part of the environmental risk factors^3–5^.

The *Betaherpesviridae* human cytomegalovirus (HCMV) is know to cause, in the immunocompetent host, an infection that usually range from asymptomatic to mild. Serious complications can however be the result of the infection of immunocompromised hosts^6^. In opposition to laboratory strains of HCMV, which growth appears to be restricted to fibroblasts only, clinical isolates are able to infect and grow in several types of cells including epithelial cells, endothelial cells, monocytes, macrophages, fibroblasts, stromal cells, hepatocytes, smooth muscle cells, and neural stem/progenitor cells^7–10^. Recently, a detailed *in vivo* evolutionary map of HCMV was built by combining population genetics methods and high throughput sequencing. This map provided evidence that viral populations under the influence of host environment can either be stable or rapidly differentiating, leading to tissue compartment colonization^11^.

Several research groups focusing on inflammatory diseases and on cancer addressed the role played by HCMV in these diseases^12–14^. Tumor tissues from several cancers, including brain, colon, prostate, liver and breast cancer, have been found positive for HCMV DNA or antigens^15–20^. In the paradigm of oncomodulation, oncogenesis mechanisms could be amplified by HCMV acting as a cofactor, when infecting the tumor tissue^21^. In addition, monocytes and macrophages, respectively located in the blood and in the tissues, may act as sites for the establishment of latency, given their role as HCMV cellular reservoirs which are responsible for the dissemination of the virus^8,22–24^. Finally, in breast carcinomas and glioblastomas, tumor-associated macrophages (TAM) are a marker of poor prognosis, and their development might be influenced by macrophage-tropic HCMV strains^25–28^. Thus the quest of new HCMV isolates which target monocytes/macrophages and thereby might play a role in oncogenesis has shown increased interest.

In the present study, we found that the genomic sequence of the HCMV-DB strain isolated from a cervical swab specimen is close to the sequence of other primary clinical isolates, especially the Toledo strain, originally isolated in the urine of a child presenting a congenital infection by HCMV, and the JP strain, isolated from a glandular tissue, the prostate^21,29^. Based on the analysis of genes involved in virus entry, our results indicate that the HCMV-DB strain is close to the Merlin strain. Given the scarcity of evidence for a direct role of HCMV in the cellular transformation of epithelial cells, we studied the transcriptome profile of HCMV-DB infected human mammary epithelial cells (HMECs). We observed that the transcriptome of HCMV-DB infected HMECs displays a triple negative ER−/PGR−/HER2− phenotype, presents some oncogenic traits, favors cell cycling and cell proliferation, and modulate angiogenesis and proteolysis. All these phenomena are potentially involved in tumor development. Finally, the infection of HMECs with the HCMV strain DB resulted in enhanced proliferation and tumorsphere formation *in vitro*.

## Results

### Genomic profile of the HCMV-DB strain

**Figure 1:**
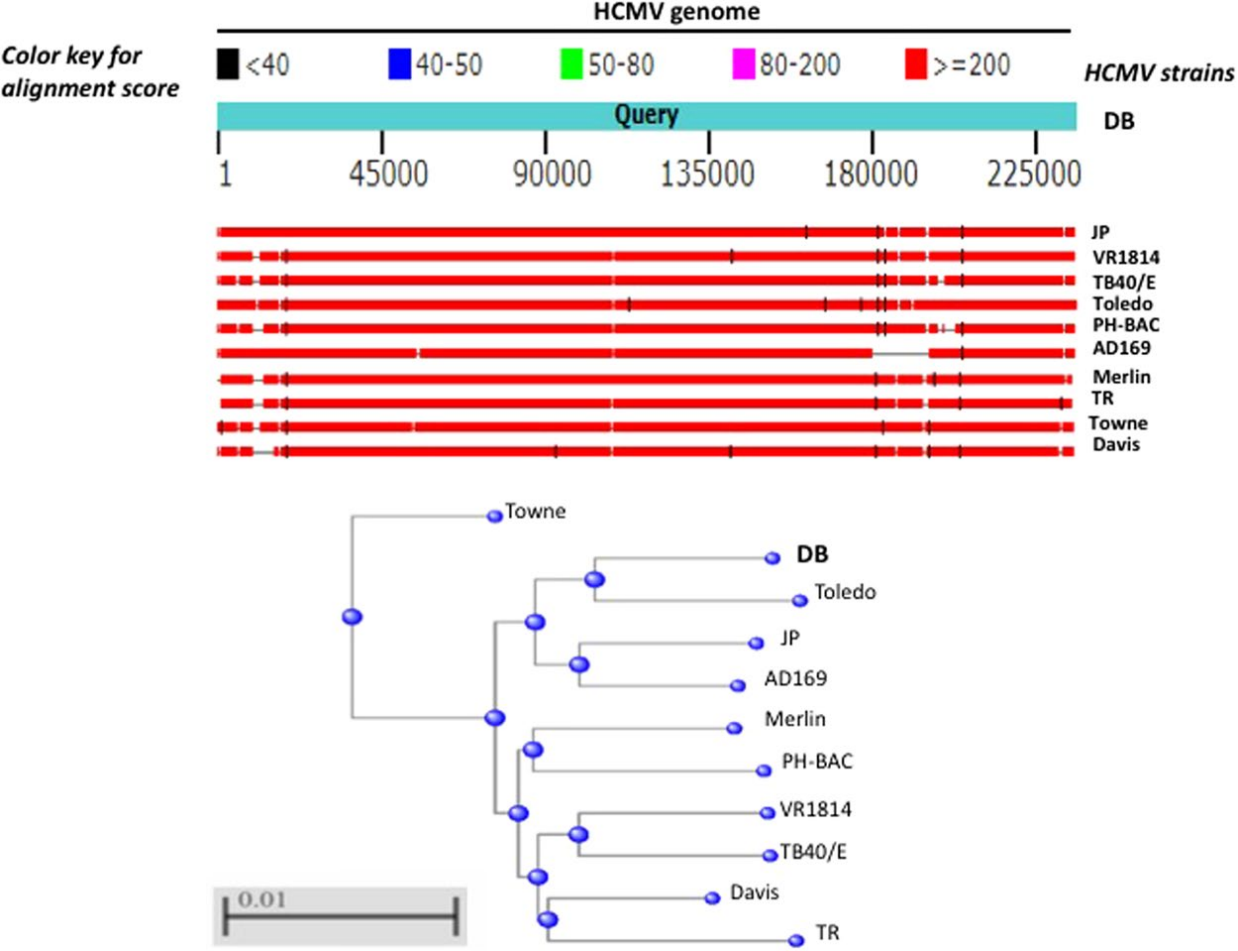
Comparison of the genomic sequence of HCMV-DB with the genomic sequences of other clinical and laboratory adapted HCMV strains. The upper panel shows the genomic sequence of HCMV-DB as compared to two laboratory adapted strains (AD169 and Towne) and eight low passages clinical isolates (Merlin, Toledo, TR, PH, VR1814, Davis, JP, and TB40/E). The lower panel shows the alignment tree related to the whole viral genome with comparison between HCMV-DB genome and the genome of ten other HCMV strains.

We previously isolated a novel HCMV strain, that we characterized and named HCMV-DB^8^. We compared its genomic sequence to that of ten HCMV strains, including two laboratory adapted strains (AD169, Towne) and eight clinical isolates with low passages in culture (Merlin, Toledo, TR, PH, VR1814, Davis, JP, TB40/E)^30,31^. The complete genomic sequence of HCMV-DB has a total length of 235,512 bp (Fig. 1 and Table 1). In comparison the sequence of the Merlin strain has approximately the same size (235,645 bp), the laboratory adapted strain AD169 has the shortest sequence (229,354 bp) and the clinical strain TB40/E the longest sequence (237,683 bp) (Fig. 1 and Table 1). Clinical isolates with low passages in culture have a total length ranging from 229,700 bp for PH to 237,683 bp for TB40/E (Fig. 1 and Table 1).

**Table 1:** Genomic comparison of HCMV-DB with other HCMV strains.

HCMV strain	Clinical	Geographical	DNA	Passages	Nucleotide length	Genes Mutated	Max. score (e + 05)	Total score (e + 05)	Query cover	Identity	Accession
DB	Pregnant women	France	Clinical material on macrophage cultures	low passage on fibroblasts	235512	RL13, UL9			100	100	KT959235
Towne	Urine from a congenitally infected enfant	USA	Fibroblast culture cells	many	235147	many: RL13, UL1, UL40, UL130, US1	1.316	3.929	95	99	FJ616285
AD169	Adenoid tissue	USA	Fibroblast culture	many	229354		1.238	4.036	92	98	X17403.1
DAVIS	Liver biopsy from a congenitally infected infant	USA	Fibroblast culture	many	229768	RL5A, RL12, RL13, UL1, UL2, UL4, UL5, UL6, UL99, UL130	1.29	3.936	95	98	JX512198.1
JP	Post mortem prostate tissue from an AIDS patient	UK	Clinical material	low passage on fibroblasts	236375		2.838	4.129	98	99	GQ221975.1
Merlin	Urine from a congenitally infected infant	UK	Clinical material	low passage on fibroblasts	235646	RL13	2.841	4.034	97	98	AY446894
PH	Bone marrow transplant recipient	USA	Clinical material	low passage on fibroblasts	229700		1.545	3.856	95	98	AC146904
TB40/E	Throat wash of a bone marrow transplant recipient	Germany	Fibroblast culture	Few	237683	RL13, UL128, IRS1, US1, US2	1.555	3.962	96	98	KF297339
Toledo	Urine from a congenitally infected infant	USA	Fibroblast culture	several	235404	RL13, UL9, UL128	1.548	4.171	99	98	GU937742
TR	Vitreous humor from eye of HIV-positive male	USA	Fibroblasts	several	235681		1.539	3.955	96	98	KF021605.1
VR1814 (FIX)	Cervical secretions of a pregnant woman with a primary HCMV infection	Italy	Clinical material	low passage on fibroblasts	235233		1.564	3.995	97	98	GU179289

Based on the sequence similarity between HCMV strains, the highest total scores by Blast were measured for JP and Toledo strains when compared to the DB strain (Table 1). The DB strain shares around 99% identity with JP and Toledo strains, by linear full genome alignment (Table 1). The other clinical isolates analyzed share 98% identity with the DB strain (Table 1). HCMV-DB strain is mutated in RL13 and UL9 genes, but not in the ULb’ region (Table 1). Altogether our analyses indicate that the genomic sequence of HCMV-DB is highly similar to the genomic sequences of the clinical strains JP and Toledo.

### Phylogenetic classification of the HCMV-DB strain based on genes involved in virus entry

**Figure 2:**
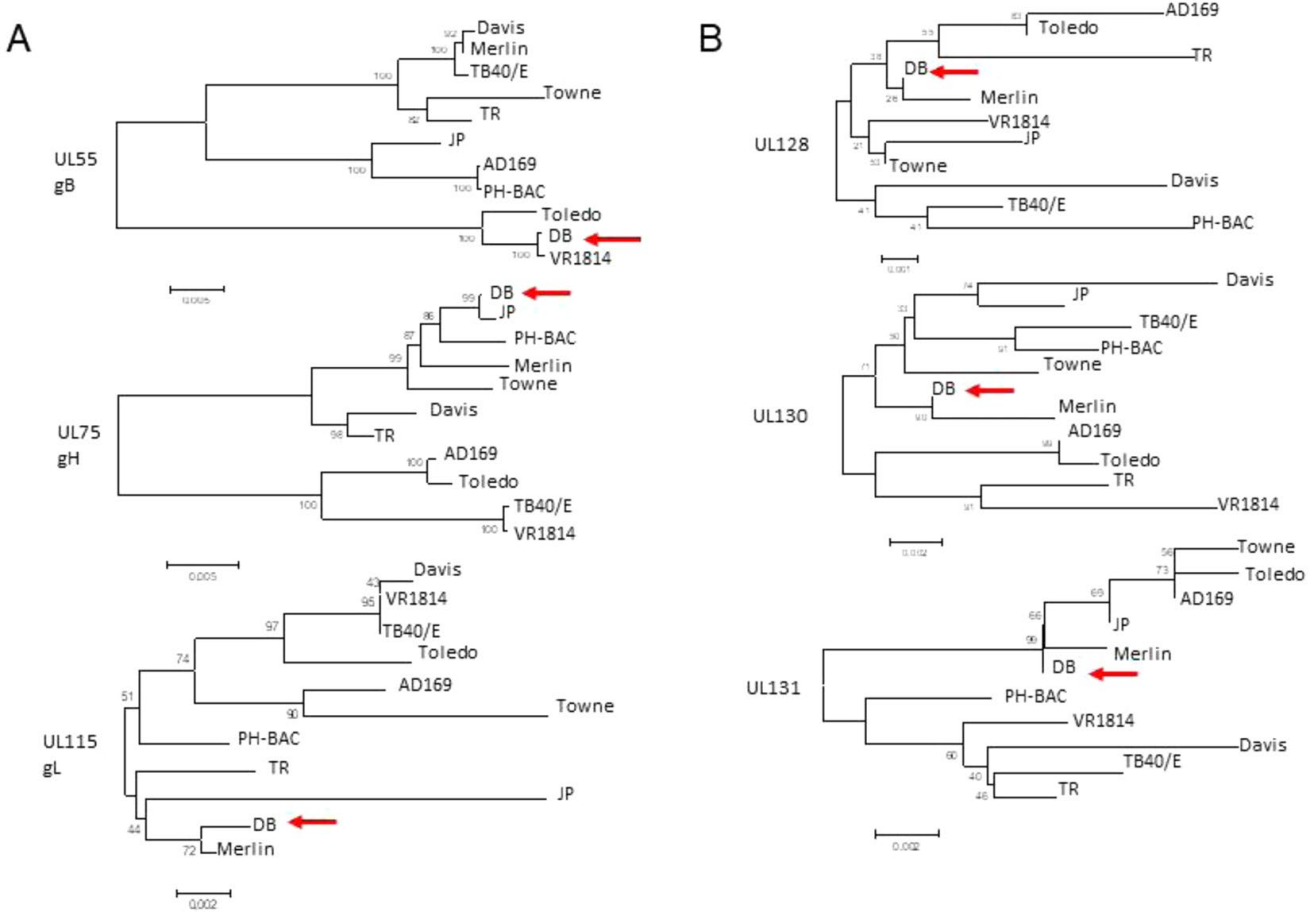
Phylogenetic analyses comparing HCMV-DB with several HCMV strains for genes involved in viral entry. (**A**) Phylogenetic analysis of genes coding for viral glycoproteins required for the entry into cells: UL55 (gB), UL75 (gH), and UL115 (gL). (**B**) Phylogenetic analysis of genes coding for UL128, UL130, and UL131 required in addition to the envelop glycoproteins for the viral entry into monocytes, macrophages, epithelial cells, and endothelial cells.

Since HCMV-DB is a highly macrophage-tropic strain^8^, we performed a phylogenetic analysis to compare the genes coding for the viral envelop that are involved in tropism (Fig. 2, Suppl. Table 1)^32–34^. For entry into fibroblasts, HCMV only requires the minimal complex of gB:gH/gL^32,35^. For entry into monocytes, macrophages, epithelial cells, and endothelial cells, HCMV requires, in addition to gB, a pentameric complex formed by glycoproteins gH and gL and proteins pUL128, pUL130 and pUL131^7,36–41^. We observed that the gB:gH/gL sequences from DB are close to those of VR1814, JP and Merlin, respectively (Fig. 2A). The HCMV-DB pentameric complex was close to that of JP for gH, and to that of Merlin for gL, UL128, UL130 and UL131 (Fig. 2A,B). For gB genotype, the DB strain is close to the VR1814 strain (Fig. 2A). Taking into account the sequences of the genes involved in virus entry, altogether our phylogenetic analysis indicates that the HCMV-DB strain is close to the Merlin strain. The UL144 gene, situated in the ULb’ region of the viral genome, is commonly used for genotyping HCMV and we previously reported that HCMV-DB belongs to the UL144 genotype C^29^.

### HCMV-DB infects HMECs *in vitro*

**Figure 3:**
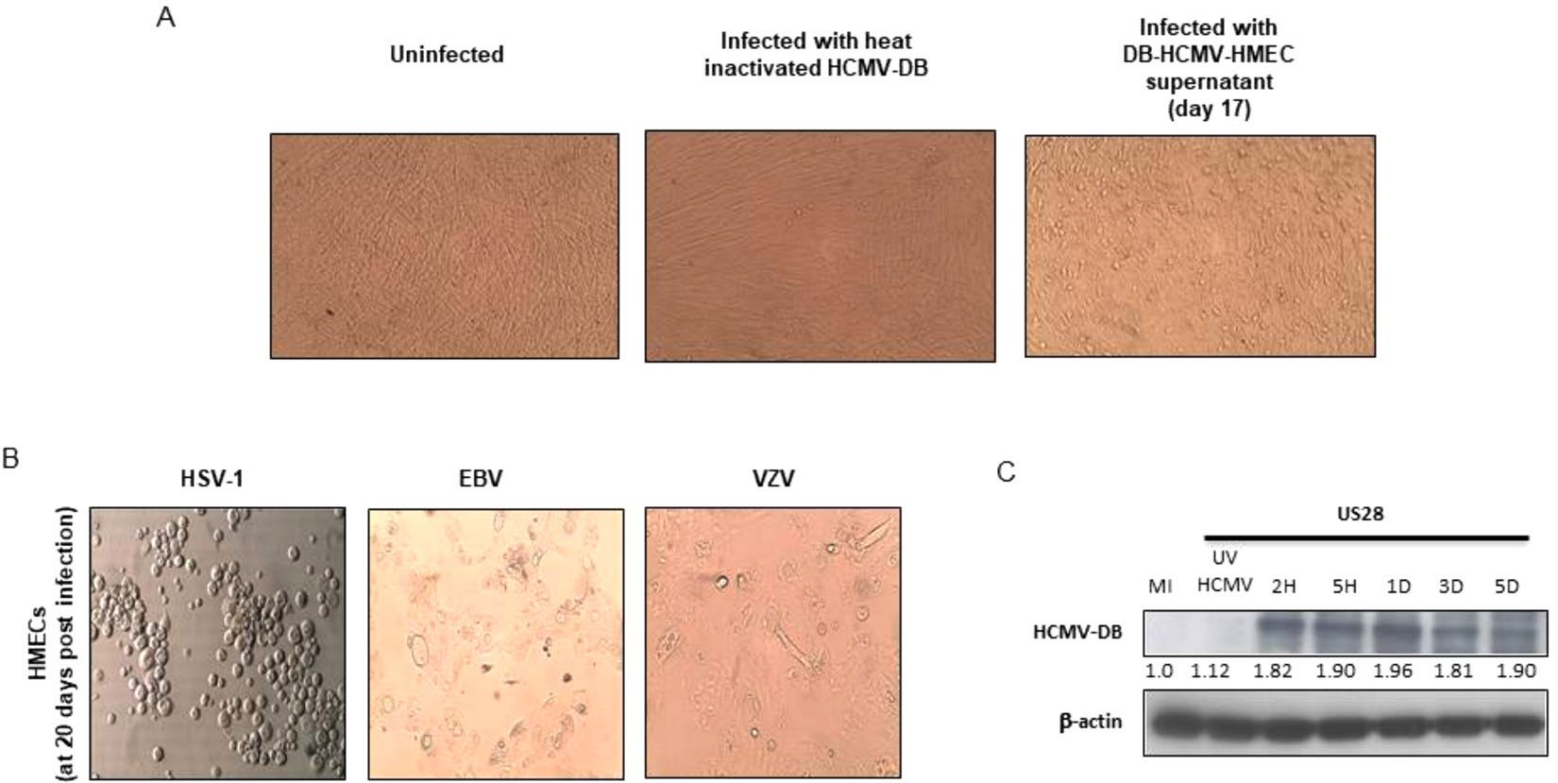
HCMV-DB infects HMECs. (**A**) HCMV cytopathic effect (CPE) in MRC5 cultures infected with supernatants harvested from HCMV-DB infected HMECs. Heat inactivated HCMV-DB is used as a negative control. (**B**) Clusters of round cells in HMECs infected with HSV-1. No CPE detected with the direct infection of HMECs with EBV and VZV. (**C**) Western blotting showing the time-course expression of pUS28 in HCMV-DB infected HMECs.

To determine whether HMECs are permissive to HCMV-DB, HMECs were infected with HCMV-DB. We harvested the supernatants of infected HMECs 17 days post infection and added it to MRC5 cells. We observed a cytopathic effect (CPE) typical of HCMV in MRC5 cultures infected with these supernatants (Fig. 3A). We did not detect any CPE in HMECs directly infected with Epstein-Barr virus (EBV) and varicella zoster virus (VZV) (Fig. 3B). In contrast, direct infection of HMECs with herpes simplex virus type 1 (HSV-1) resulted in the appearance of clusters of round refringent cells (Fig. 3B). To further confirm the direct infection of HMECs by HCMV-DB we detected the early protein pUS28 using western blotting in HCMV-DB infected HMECs after 2 hours and up to 5 days post infection (Fig. 3C). Our results confirm our previously published data that indicate a full replicative viral cycle of HCMV-DB in HMECs^29^.

### The transcriptome of HCMV-DB infected HMECs displays a triple negative basal-like phenotype

Uninfected HMECs and HCMV-DB-infected HMECs at low and high MOIs (1 or 10) were used one day after infection for the screening with oncogenes/tumor suppressor genes and human breast cancer genes RT^2^ profiler PCR assays (PAHS-502Z and PAHS-131Z, respectively). Positive controls included MCF-7 and MDA-MB231 breast cancer cell lines as luminal and basal-like phenotypes, respectively. Each of the RT^2^ Profiler PCR arrays profiles the expression of 84 key genes -which overlap sometimes in the two assays- commonly involved in tumor classification, signal transduction, and other commonly affected pathways such as angiogenesis, adhesion, proteolysis, cell cycle, and apoptosis (Suppl. Tables 2 and 3).

**Figure 4:**
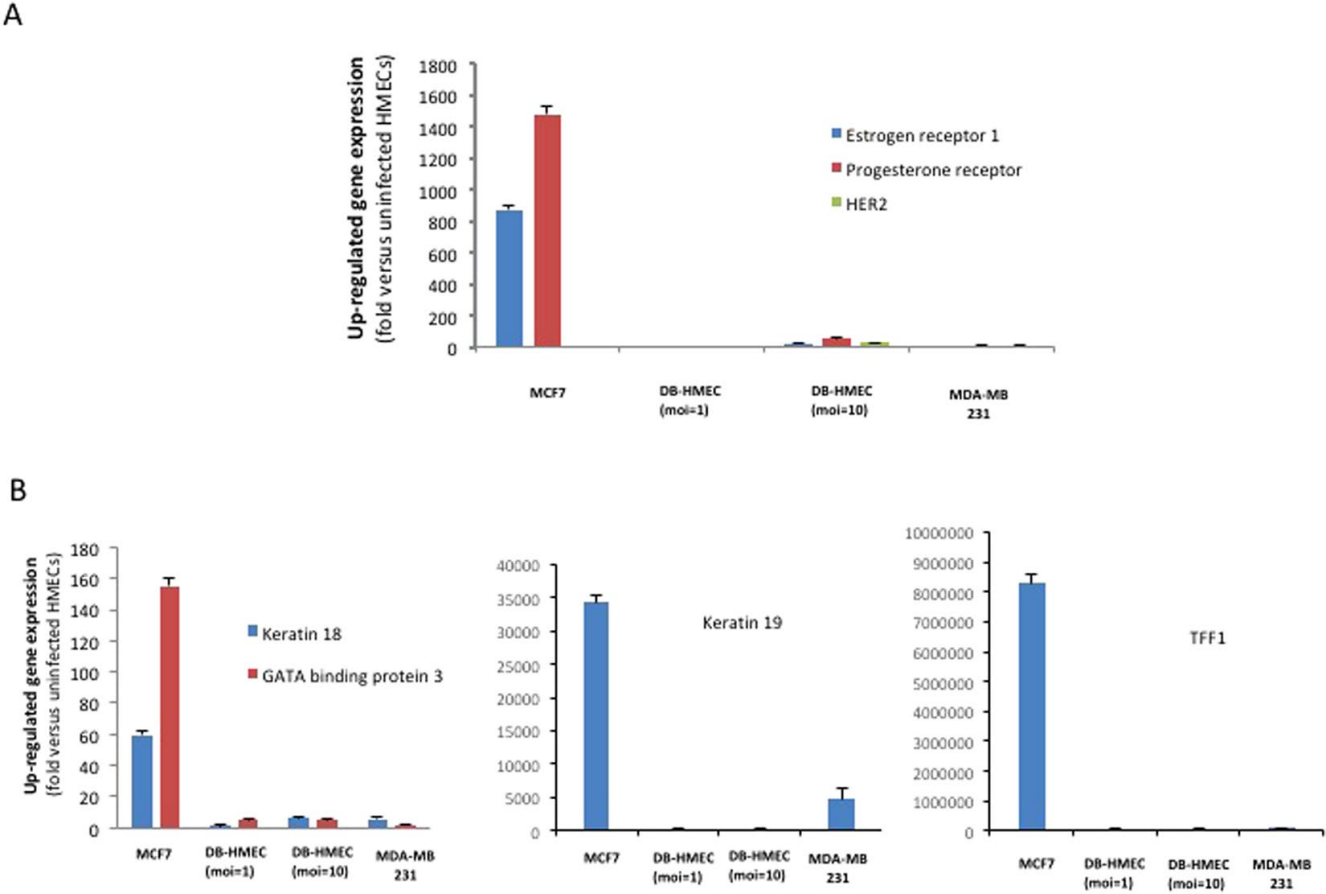
Transcriptome analysis of HMECs infected with HCMV-DB displays a triple negative basal-like phenotype. (**A**) HMECs infected with HCMV-DB and MDA-MB-231 cells show a similar pattern of ER−/PGR−/HER2− transcripts, in contrast to the ER+/PGR+/HER2− transcripts detected in MCF-7 cells. (**B**) Low levels of gene expression of luminal markers (KRT18, KRT19, GATA3, TFF1) in both HCMV-DB infected HMECs and MDA-MB-231 cells as compared to the luminal MCF-7 cells.

In regard to the expression of ER/PGR/HER2 transcripts, HCMV-DB infected HMECs and MDA-MB231 cells showed a similar transcriptome that was clearly distinct from the transcriptome of MCF-7 cells (Fig. 4A). Although the triple negative ER−/PGR−/HER2− phenotype was present in both infected and uninfected HMECs, the levels of estrogen receptor (ESR1), progesteron receptor (PGR) and HER2 (ERBB2) transcripts were higher in HCMV-DB infected HMECs in comparison to uninfected HMECs (Fig. 4A). The gene expression of luminal markers (KRT19, KRT18, GATA3, TFF1) was low in HCMV-DB-infected HMECs, similar to the phenotype of MDA-MB231 cells (Fig. 4B, Suppl. Tables 2 and 3). In contrast, MCF-7 cells expressed high levels of luminal markers transcripts (Fig. 4B).

### The transcriptome of HMECs infected with HCMV-DB presents oncogenic traits with enhanced cellular proliferation

**Figure 5:**
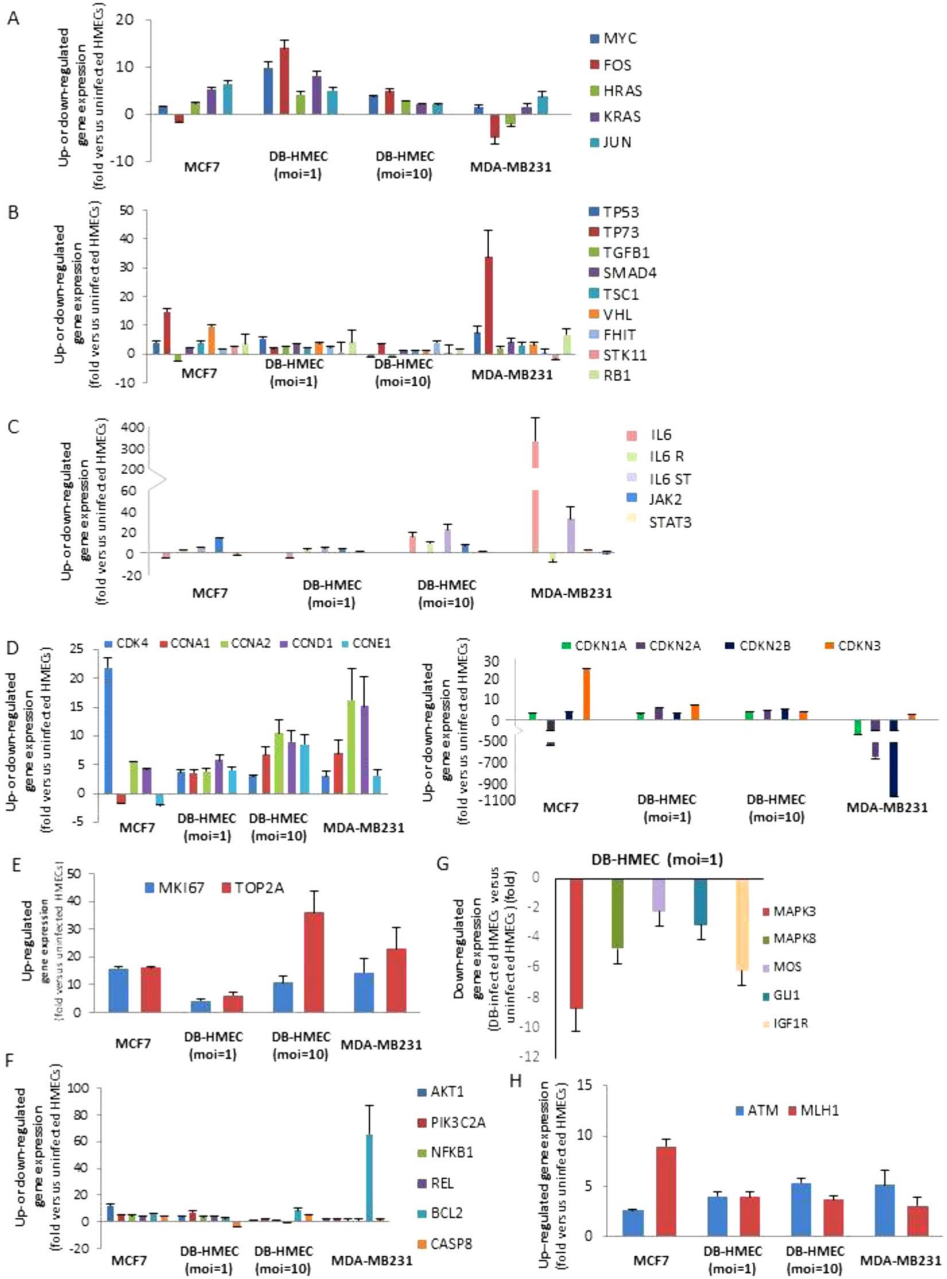
The transcriptome of HMECs infected with HCMV-DB displays oncogenic traits. (**A**) Upregulation of the gene expression of oncogenes: Myc (MYC), Fos (FOS), Jun (JUN), KRas (KRAS), HRas (HRAS) and NRas (NRAS), (**B**) Upregulation of the gene expression of tumor suppressor genes: TP53, TP73, FHIT, VHL, SMAD4, TGFB1, STK11, TSC1, RB1. (**C**) Expression of genes from the IL-6/JAK-STAT3 axis. (**D**) *Left panel*, Upregulation of genes coding for the cyclins: CCNA1, CCNA2, CCND1, CCNE1 and the cyclin dependent kinase 4 CDK4. *Right panel*, Upregulation of genes coding for the cyclin dependent kinase inhibitors: CDKN 1 A, CDKN 2 A, CDKN 2B and CDKN 3. (**E**) Upregulation of the expression of genes coding for proliferation markers, the Ki67 antigen (MKI67) and the topoisomerase 2 (TOPO2A). (**F**) Upregulation of the expression of genes involved in cell survival (NFKB1, REL, AKT1, PIK3C2A, BCL-2) and downregulation of the expression of caspase 8 (CASP8). (**G**) Downregulation of gene expression of the members of the MAPK cascade (MAPK3, MAPK8, MOS, GLI1, IGF1R). (**H**) Upregulation of the expression of genes involved in DNA reparation: the ataxia telangiectasia mutated (ATM) and human MutL homolog (MLH1). The up- and down-regulation were measured in HMECs infected with HCMV-DB (moi, 1 and 10) as compared to uninfected HMECs.

The gene expression of the oncogenes Myc (MYC), Fos (FOS), Jun (JUN), KRas (KRAS), HRas (HRAS) and NRas (NRAS) is upregulated in HCMV-DB-infected HMECs in comparison to uninfected HMECs (Fig. 5A). We also observed the upregulation of transcripts of numerous other oncogenes (KITLG, MCL1, MET, MYB, NFKBIA, PIK3CA, PML, PRKCA, RAF1, RARA, ROS1, RET, ABL1, ETS1, RUNX1, RUNX3) (Suppl. Tables 2 and 3). The gene expression of the tumor suppressor genes coding for the retinoblastoma protein (RB1) and the p53 protein (TP53) was upregulated in HCMV-DB-infected HMECs, as reported previously for basal-like triple negative cell lines such as MDA-MB231 cells (Fig. 5B and Suppl. Table 3)^42,43^. Similarly, the expression of several other tumor suppressor genes (TP73, FHIT, VHL, SMAD4, TGFB1, STK11, TSC1) is also upregulated in HCMV-DB-infected HMECs, mostly at low moi (moi = 1) compared to uninfected HMECs (Fig. 5B).

The IL-6/JAK-STAT3/Cyclin D1 axis is activated in biopsies from breast cancer patients^44^. We observed the upregulation of IL-6, IL-6 receptor and JAK2 gene expression in HCMV-DB infected HMECs compared to uninfected HMECs especially at high moi (moi = 10), with stable levels of STAT3 gene expression (Fig. 5C). The gene expression of cyclin dependent kinase 4 (CDK4) and of several cyclins (CCNA1, CCNA2, CCND1, CCNE1) especially cyclin D1 which function as regulator of CDKs is up-regulated in HCMV-DB-infected HMECs (moi = 10) compared to uninfected controls (Fig. 5D, left panel). The gene expression of CDK inhibitors (CDKN) 1A (p21), 2A (p16), 2B (p15), and 3 (CDKN1A, CDKN2A, CDKN2B, CDKN3) is up-regulated by 5 folds in HCMV-DB-infected HMECs compared to uninfected cells (Fig. 5D, right panel).

In agreement with activation of the IL6/JAK/cyclin D1 pathway in HCMV-DB-infected HMECs, we observed the upregulation expression of proliferation marker genes such as the Ki67 antigen gene (MKI67) and the topoisomerase 2 gene (TOPO2A) when compared to uninfected HMECs (Fig. 5E). The expression of genes involved in cell survival (NFKB1, REL, AKT1, PIK3C2A, BCL-2) is increased in HCMV-DB-infected HMECs compared to uninfected HMECs, indicating a prosurvival signal in infected cells (Fig. 5F). In agreement with the induction of several prosurvival genes in infected cells compared to uninfected controls, the down-regulation of caspase 8 transcript (CASP8) is observed in HCMV-DB-infected HMECs at moi 1 compared to uninfected cells (Fig. 5F).

### The transcriptome of HCMV-DB infected HMECs displays modifications in cell signaling, angiogenesis and proteolysis

The gene expression of the members of the MAPK cascade (MAPK3, MAPK8, MOS, GLI1, IGF1R) is down-regulated in HCMV-DB-infected HMECs (Fig. 5G). The gene expression for the transcription factors, tumor suppressor gene hypermethylated in cancer 1 (HIC1) and forkhead box D3 (FOXD3) is up-regulated in HCMV-DB-infected HMECs at high moi (Suppl. Figure 1). In HCMV-DB-infected HMECs, we observed the upregulation of the expression of the ataxia telangiectasia mutated (ATM) and human MutL homolog (MLH1) genes, both involved in DNA reparation (Fig. 5H).

**Figure 6:**
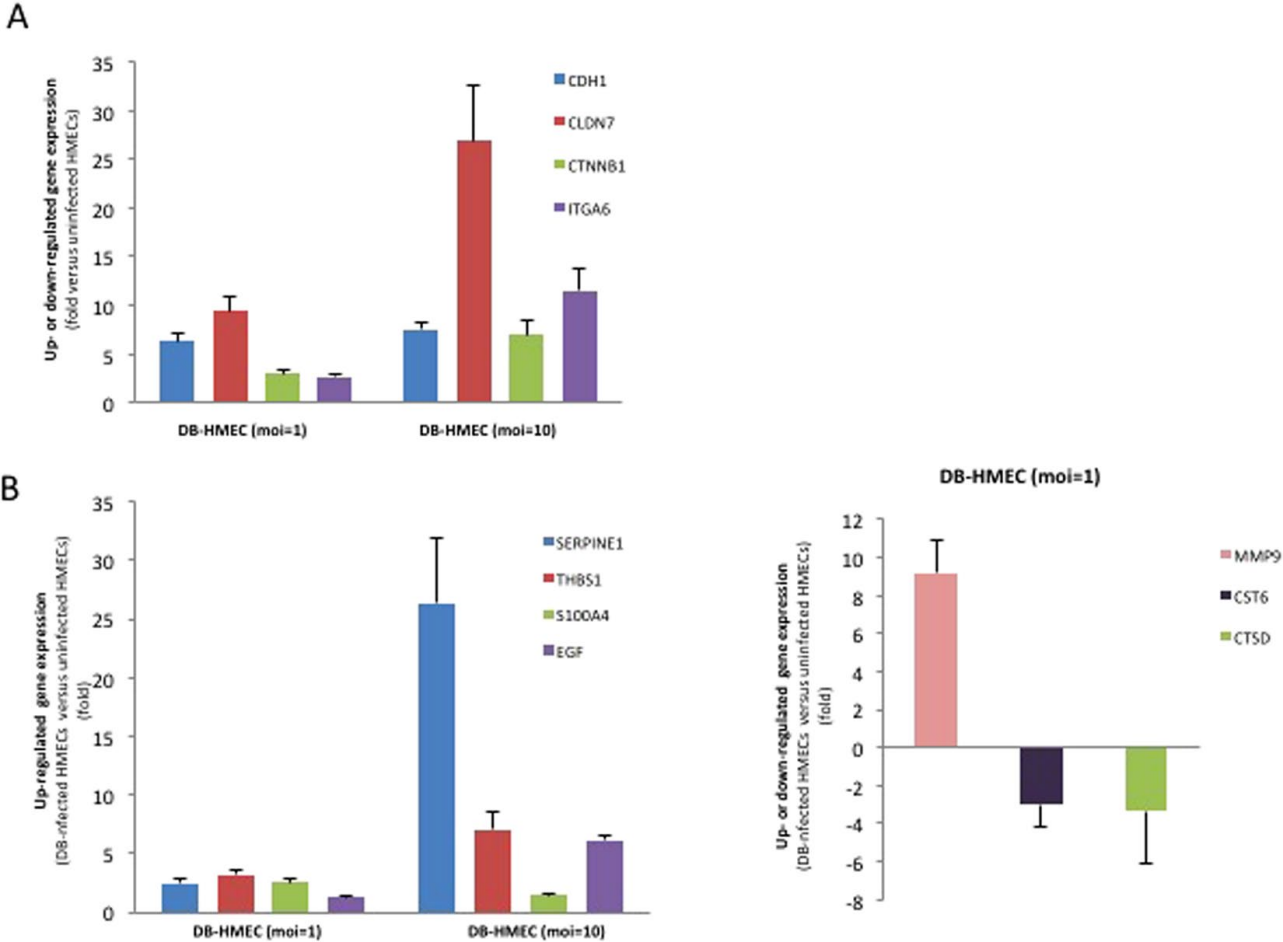
Modification of the transcriptome of genes involved in cell adhesion, angiogenesis and proteolysis in HMECs infected with HCMV-DB. (**A**) Upregulated gene expression of E-cadherin (CDH1), claudin 7 (CDN7), beta-catenin (CTNNB1), alpha 6 integrin (ITGA6); (**B**) Upregulated expression of genes involved in angiogenesis (SERPINE1, THBS1, S100A4, and EGF) and proteolysis (MMP9), and downregulated expression of genes involved in proteolysis: CST6 and CTSD. The up- and down-regulation were measured in HMECs infected with HCMV-DB (moi, 1 and 10) as compared to uninfected HMECs.

The gene expression of E-cadherin (CDH1), claudin 7 (CLDN7), beta-catenin (CTNNB1) and alpha 6 integrin (ITGA6) is upregulated in HCMV-DB-infected HMECs at high moi (moi = 10) (Fig. 6A). In HCMV-DB-infected HMECs, we observed both the up- and downregulation of genes involved in angiogenesis (upregulation: IL-6, SERPINE1, THBS1, S100A4, EGF; downregulation: SLIT2) and proteolysis (upregulation: MMP9; downregulation: CST6, CTSD) (Fig. 6B, Suppl. Table 3).

### The transcriptome of HMECs infected with HCMV-DB reveals a global hypomethylation state

**Figure 7:**
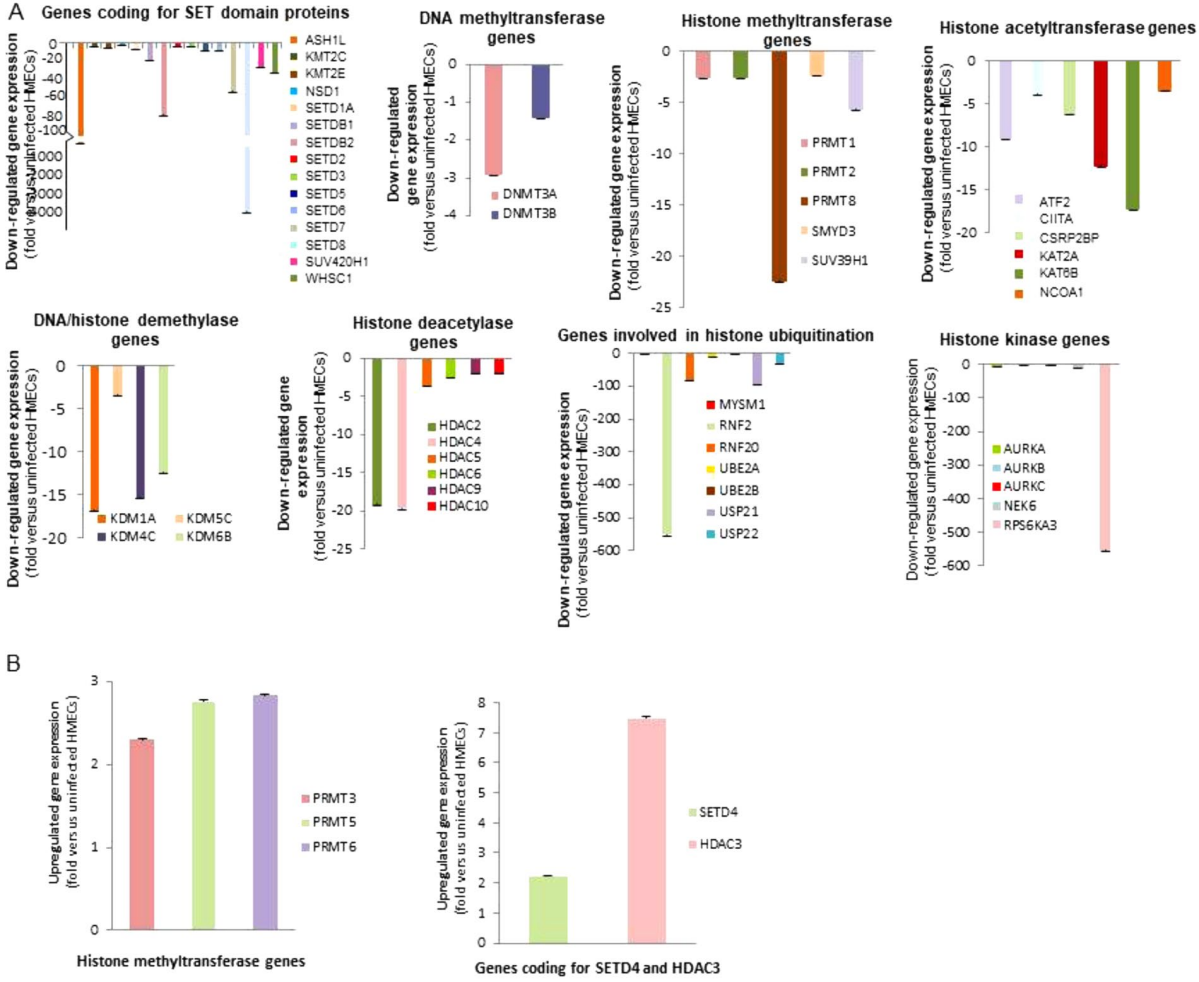
A general hypomethylation state was observed in HMECs infected with HCMV-DB. (**A**) Downregulation of the gene expression of DNA methyltransferase genes (DNMT3A), histone methyltransferase genes (PRMT1, PRMT2, PRMT8, SMYD3, SUV39H1), genes coding for SET domain proteins with a histone methyltransferase activity (ASH1L, MLL3 (KMT2C), MLL5 (KMT2E), NSD1, SETD1A, SETDB1, SETDB2, SETD2, SETD3, SETD5, SETD6, SETD7, SETD8, SUV420H1, WHSC1), histone acetyltransferase genes (ATF2, CIITA, CSRP2BP, KAT2A, KAT6B, NCOA1), histone kinase genes (AURKA, AURKB, AURKC, NEK6, RPS6KA3), genes involved in histone ubiquitination (MYSM1, RNF2, RNF20, UBE2A, UBE2B, USP21, USP22), DNA/histone demethylase genes (KDM1A, KDM5C, KDM4C, KDM6B) and histone deacetylase genes (HDAC2, HDAC4, HDAC5, HDAC6, HDAC9, HDAC10). (**B**) Upregulation of the gene expression of histone methyltransferase genes (PRMT3, PRMT5, PRMT6), genes coding for SET domain proteins with a histone methyltransferase activity (SETD4) and HDAC3. The up- and down-regulation were measured in HMECs infected with HCMV-DB (moi, 1 and 10) as compared to uninfected HMECs.

Since epigenetic regulation of chromatin has been associated with breast cancer classification and prognosis, we performed a Human Epigenetic Chromatin Modification Enzymes RT^2^ Profiler PCR Array (PAHS-085A) which profiles the expression of 84 key genes encoding enzymes known or predicted to regulate chromatin accessibility, and therefore gene expression, by modifying genomic DNA and histones (Suppl. Tables 4 and 5). Considering a standard two-fold increase or decrease of RNA expression between HCMV-DB-infected HMECs and uninfected HMECs to be biologically significant, performed analysis indicated the down-regulation of most (n = 50) of the genes including DNA methyltransferase genes (DNMT3A, DNMT3B), histone methyltransferase genes (PRMT1, PRMT2, PRMT8, SMYD3, SUV39H1), genes coding for SET domain proteins with a histone methyltransferase activity (ASH1L, MLL3 (KMT2C), MLL5 (KMT2E), NSD1, SETD1A, SETDB1, SETDB2, SETD2, SETD3, SETD5, SETD6, SETD7, SETD8, SUV420H1, WHSC1), histone acetyltransferase genes (ATF2, CIITA, CSRP2BP, KAT2A, KAT6B, NCOA1), histone kinase genes (AURKA, AURKB, AURKC, NEK6, RPS6KA3), genes involved in histone ubiquitination (MYSM1, RNF2, RNF20, UBE2A, UBE2B, USP21, USP22), DNA/histone demethylase genes (KDM1A, KDM5C, KDM4C, KDM6B) and histone deacetylase genes (HDAC2, HDAC4, HDAC5, HDAC6, HDAC9, HDAC10) (Fig. 7A, Suppl. Table 4), and the upregulation of few genes (n = 5) including histone methyltransferase genes (PRMT3, PRMT5, PRMT6), a gene coding for SET domain proteins with a histone methyltransferase activity (SETD4) and the histone deacetylase HDAC3 (Fig. 7B, Suppl. Table 4). Altogether our results indicate an overall down-regulation of transcripts involved in methylation of DNA and histones in HCMV-DB-infected HMECs. In addition, transcripts of histone acetyltransferase and deacetylase genes are mostly downregulated in HCMV-DB-infected HMECs compared to uninfected controls.

### Formation of tumorspheres from HMECs infected with HCMV-DB

**Figure 8:**
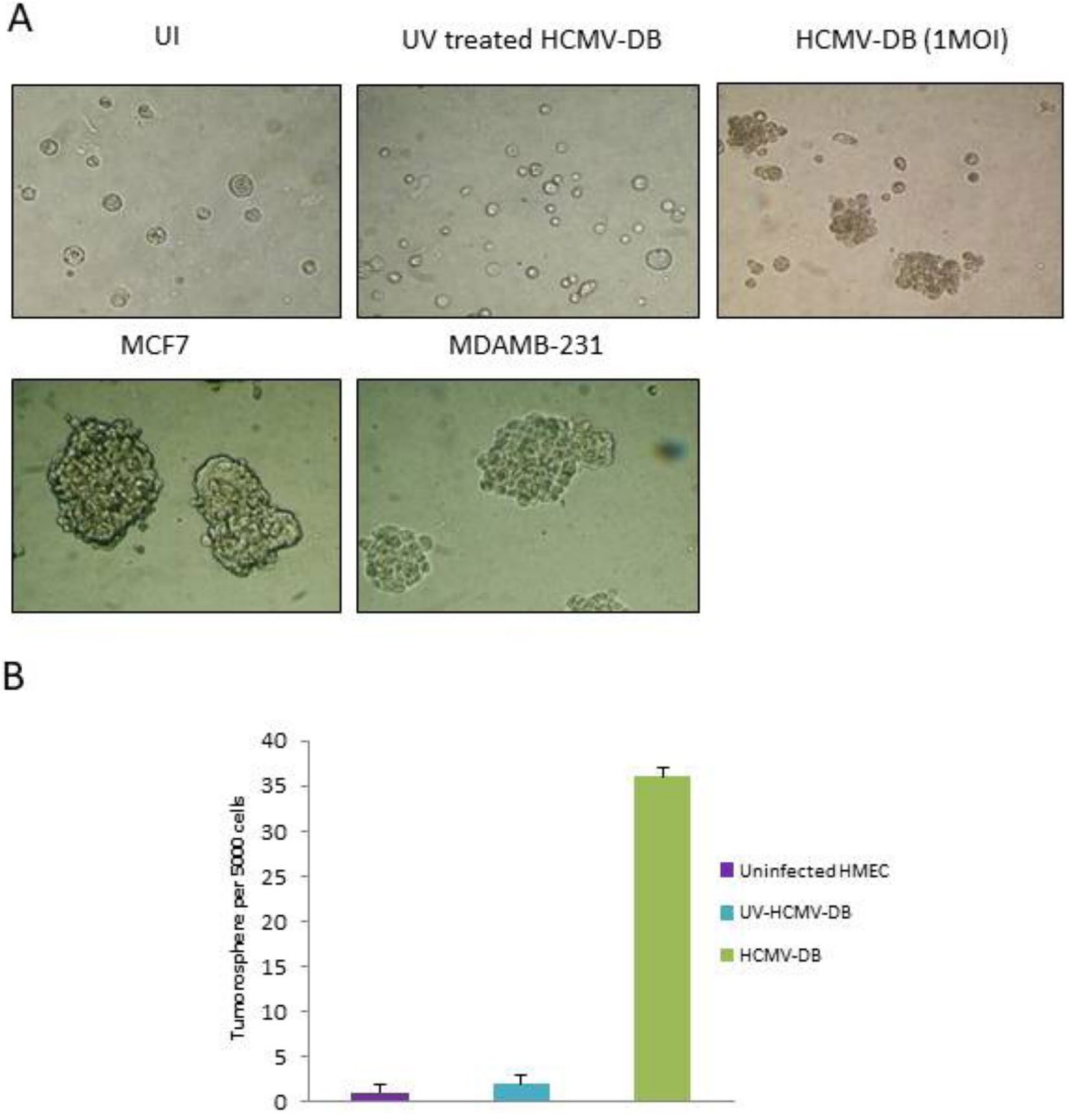
HMECs infected with HCMV-DB show tumorspheres formation. (**A**) Tumorsphere formation in HMECs infected with HCMV-DB as compared to uninfected HMECs (UI) and HMECs infected with UV-treated HCMV-DB. MCF-7 and MDA-MB-231 cells are used as positive controls. (**B**) The histogram represents quantification of tumorsphere formation in the cultures of HMECs infected with HCMV-DB. Uninfected HMECs and HMECs infected with UV-inactivated HCMV-DB were used as negative controls. Results are means (±SD) of three independent experiments.

Since it was previously showed that the activation of the IL-6/STAT3 axis signaling in cancer stem cells (CSC) can enhances proliferation and survival of cells and in turn favor the growth of tumors in mice, we decided to detect the presence of CSC in HCMV-DB-infected HMECs using a tumorsphere (mammosphere) formation assay^45,46^. To determine whether the induction of CSC expansion could be the result of the HCMV-DB infection of HMECs, we infected them for 1 day and evaluated the proportion of stem-like cells by sphere formation assay. We found that HCMV-DB infected cultures formed tumorspheres (Fig. 8A,B). As expected MCF-7 cells and MDA-MB231 cells formed tumorspheres (Fig. 8A). In contrast, the use of UV-inactivated HCMV-DB to infect HMECs did not result in tumorspheres formation in cultures, neither did the use of uninfected HMECs (Fig. 8A,B).

## Discussion

Our results indicate that, based on its genomic profile, the HCMV-DB strain is similar to clinical HCMV strains especially the JP and Toledo strains. Based on phylogenetic analysis of genes involved in virus entry, the HCMV-DB strain is close to the Merlin strain. In addition, we observed that HCMV-DB infects HMECs which display a triple negative phenotype. The transcriptome of HCMV-DB-infected HMECs shows the characteristics of a pro-oncogenic cellular environment with upregulated expression of numerous oncogenes, enhanced activation of pro-survival genes, and upregulated markers of cell proliferation, stemcellness and EMT that was confirmed by enhanced tumorsphere formation *in vitro*.

HCMV clinical isolates can infect among others epithelial cells, endothelial cells, monocytes, macrophages, fibroblasts, stromal cells, hepatocytes, smooth muscle cells, and neural stem/progenitor cells. In opposition to this broad cellular tropism, HCMV laboratory strains can only grow on fibroblasts^7^. Some clinical HCMV isolates infect the blood monocytes and tissue macrophages, induce a distinct macrophage polarization and establish latency in monocytes/macrophages^8,22,24,47^. The tropism of HMCV strains could have a major role in M1/M2 macrophage activation and enhance the viral fitness, ultimately favoring breast cancer promotion^26,48,49^. Previously, we isolated the HCMV-DB strain (KT959235)^8^. The HCMV-DB strain, given its characterization as highly macrophage-tropic, induces an M2 polarization of the macrophages and the upregulation of the proto-oncogene Bcl-3^8^. The ULb’ region of the HCMV-DB strain is intact^29^ and HCMV-DB show genetic similarities to other primary clinical isolates such as the JP strain which has been isolated post mortem from the prostate of an HIV-infected patient^30,31^. Interestingly, DB and JP strains infect mammary and prostate glands respectively which show numerous similarities with regard to appearance, physiology and pathology^50^.

The HMECs infected with HCMV-DB exhibit a triple negative ER−/PR−/HER2− phenotype with low expression of luminal markers (KRT19, KRT18, GATA3, TFF1). Oncogenic conversion of HMECs requires a limited number of changes leading towards growth deregulation^51^. These changes include the inactivation of tumor suppressor pathways, mainly p53 and retinoblastoma protein (Rb)^52^, the establishment of telomere maintenance^53,54^, the activation of the Akt pathway and activation of mitogenic signal delivered by oncogenes such as Myc and Ras^51,55,56^. We observed the upregulation of Rb and p53 transcripts in HCMV-DB-infected HMECs. Increased p53 transcript and protein expressions have been reported in fibroblasts infected with several HCMV strains^57^. The functional inactivation of p53 required for cell transformation, could be explained by the previously reported potential inhibition of the transcriptional activity of p53 by HCMV-IE2-86^58^. In agreement with the upregulation of the p53 transcript, we previously reported that, in HCMV-DB infected HMECs, the p53 protein expression was enhanced, as well as its binding to IE2 and therefore could explain its functional inactivation^29^. The gene expression of retinoblastoma protein (pRb) is 7 fold higher in HCMV-DB-infected HMECs in comparison to uninfected HMECs at day 1 post infection. We also observed the upregulation of the Rb transcript in MDA-MB231 cells in agreement with the upregulation of the pRb protein in MDA-MB231 cells reported previously^42^. Although the expression of the gene Rb is increased in HCMV-DB-infected HMECs, the post translational phosphorylation of pRb has been reported to play a critical role in its inactivation^56^. The HCMV UL97 protein (pUL97) exhibits similar activities to that of cellular cyclin-dependant kinase (CDK) complexes; thus it phosphorylates and inactivates the pRb protein. The pUL97 protein lacks the amino acid residues conserved in CDKs that allow for the attenuation of kinase activity, and is not inhibited by the CDK inhibitor p21. Therefore the activity of pUL97 is immune from normal CDK control mechanisms^59,60^. Also the human cytomegalovirus pp71, encoded by UL82, has a role in the degradation of the retinoblastoma family of proteins^61^. In agreement with such an hypothesis, we reported previously the upregulation of UL82 transcript and protein (pp71) at day 1 and 3 post infection in HMECs infected with HCMV-DB^29^. Thus, in HCMV-DB infected HMECs, both p53 and pRb proteins could be inactivated, most likely blocking cellular senescence, and therefore promoting unchecked cell division^51^. We observed, in infected cells, the activation of IL-6/STAT3 axis with expression of the pUS28 protein, which is in agreement with other studies that confirm the role of HCMV pUS28 in mediating proliferation through this axis activation^62,63^.

The acquisition of a mitogenic signal, delivered by proto-oncogenes including c-Myc and Ras, could further allow the complete transformation of HCMV-DB-infected HMECs *in vitro*. In agrement with the enhanced expression of Myc and Ras transcripts in HCMV-DB-infected HMECs, we reported previoulsy enhanced expression of Myc and Ras proteins starting at day 1 postinfection as measured by western bloting^29^. Overexpression of other oncogene transcripts such as Fos and Jun could also participate in HMEC transformation induced by HCMV-DB^64,65^. Interestingly, the *Myc* region on chromosome 8q24.21 is a known site of frequent human papillomavirus (HPV) integration and *Myc* is overexpressed in cervical carcinoma^55,66,67^. In agreement with the upregulation of the *Myc* gene expression in HMECs infected with HCMV-DB, the activation of Fos and Myc is induced by the IE1 and IE2 proteins of HCMV^68^. In addition, *MYB* gene is induced in HCMV-DB-infected HMECs similar to enhanced *MYB* gene expression in HPV-induced carcinoma^69^. Altogether, the upregulation of oncogenes observed in HCMV-DB-infected HMECs is in part similar to the transcriptomic profile observed in HPV-induced carcinoma.

The PI3K/Akt pathway is activated in breast cancer and its activation is critical for the emergence of anchorage-independent growth, eventually leading to the transformation of HMECs^70,71^. We observed enhanced expression of genes of the PI3K/Akt pathway, in agreement with phosphorylation of Akt in HCMV-DB-infected HMECs^29^. In addition increased levels of transcripts of pro-survival genes such as Bcl-2, Akt, and NF-kB was observed parallel to decreased levels of the caspase-8 transcript in HCMV-DB-infected HMECs at low moi (moi = 1). By contrast, at high moi (moi = 10), we observed increased levels of the caspase-8 transcript in HMECs infected with HCMV-DB. This apparent discrepancy between low and high moi, could be explains as follows. At early stages of the infection after viral entry, only a limited number of virions are present within the infected cell. At this initital step of the viral life cycle anti-apoptotic mechanisms have to be activated to favor cell survival and to fuel viral replication within the infected cell. Thus it is critical that early after infection (low moi) the infected cell survives to favor the completion of the viral life cycle and to allow the production of a maximum of new progeny virions. Later during infection, when numerous virions have been produced within the infected cells (high moi), the pro-apoptotic signals e.g. enhanced caspase-8 gene expression are activated and result in the lyse of the infected cells with release of the newly produced virions. Altogether, these results indicate that the transcriptome of HCMV-DB-infected HMECs displays a flexible adaptation to the viral life cycle and can be modulated differentially depending on the intracellular viral load and/or the amount of viral input.

We observed the upregulation of gene expression of proliferation markers such as the Ki67 antigen, the topoisomerase 2 and the transcription factor E2F1 in HCMV-DB-infected HMECs compared to uninfected HMECs. Our results are in agreement with the increased proliferation previously observed in cultures of HCMV-DB-infected HMECs^29^. Parallel to enhanced cellular proliferation in HCMV-DB-infected HMECs, we noted the dysregulation of the cell cycling. In HCMV-DB-infected HMECs, we observed enhanced expression of cyclins genes (cyclin A1, D1, D2, E1) including cyclin D1, but also to a lesser extent of cell cycling inhibitors (CDKNs). In agreement with the induction of CDKN2A gene expression in HMECs infected with HCMV-DB, CDKN2A gene is strongly induced in HPV infection and is widely used as a surrogate marker for HPV carcinoma^69,72^. Although we observed enhanced proliferation of HCMV-DB-infected HMECs, we also noticed a dysregulation of cell cycling which could lead to the inhibition of the cell cycle G1 progression and to the block of G1/S transition, as previoulsy reported by other groups in fibroblasts infected with various HCMV strains^73^. In agreement with an inhibition of the cell cycle proliferation in HMECs infected with HCMV-DB, the Ras association domain-containing protein 1 (RASSF1) is upregulated in infected cells and was reported to inhibit the accumulation of cyclin D1 (Suppl. Table 3). An exquisite balance between enhanced cell cycling (enhanced cyclins gene expression) and enhanced cell cycling inhibition (enhanced CDKN gene expression), might ultimately result in enhanced cell proliferation as measured by increased Ki67Ag and TOP2A transcripts and enhanced Ki67 antigen detection in HMECs infected with HCMV-DB.

Decreased MAPK activation has been reported as pejorative in triple negative breast cancers^74^. In agreement with this observation, decreased gene expression of members of the MAPK cascades (MAPK3, MAPK8) is observed in HCMV-DB-infected HMECs which exhibit a ER−/PR−/HER2− phenotype.

Several herpesviruses have been reported to lead to genome instability with activation of the DNA reparation pathway with increased expression of the *ATM* gene^75–77^. We found that the *ATM* gene expression is upregulated in HCMV-DB-infected HMECs as reported previously in fibroblasts infected with other HCMV strains^77^. Enhanced ATM expression could favor the reparation of DNA damages such as chromosomal breaks already reported in fibroblasts infected with HCMV^78,79^, but could also lead to genome instability and thereby favor cellular transformation^80^.

Several studies, including a comprehensive study by The Cancer Genome Atlas (TCGA) Network, have shown that, in opposition to luminal estrogen receptor-positive cancers, which exhibit the highest degree of hypermethylation, the triple negative breast cancers (TNBCs) are characterized by the most extensive hypomethylation^81,82^. The expression of numerous histone methyltransferase genes and of genes coding for SET domain proteins with a histone methyltransferase activity is downregulated in HCMV-DB infected HMECs in comparison to uninfected HMECs. The gene expression of DNA methyltransferase 3 A (DMT3A) is also downregulated in infected HMECs.

Regarding mammary tumor progression, Suzuki *et al*.^83^ reported that in the tumor progression from normal mammary epithelium to ductal carcinoma *in situ* (DCIS), there is a reduction of the histone acetylation levels. This change in histone acetylation was not present when comparing DCIS to invasive ductal carcinoma. A significant decrease in histone deacetylase (HDAC) protein levels during tumor progression was also described^84^. This suggests an early role for histone acetylation modifications in breast tumor progression. We observed decreased expression of genes involved in histone acetylation (histone acetyl transferases) in HCMV-DB infected HMECs in comparison to uninfected HMECs. In addition, we observed the decreased gene expression of several histone deacetylases, including HDAC2, 4, 5, 6, 9 and 10 in HCMV-DB-infected HMECs in comparison to uninfected HMECs. Altogether, our data indicate that HCMV-DB-infected HMECs at day 1 post infection display already alterations of histone acetylation which have been evoked as an early event in breast tumor progression.

The transcriptomic analysis of HCMV-DB-infected HMECs indicates that genes involved in angiogenesis and proteolysis, and thereby potentially participating in the remodelling of the tumor microenvironment, are modulated in HCMV-DB-infected HMECs^85,86^. We also observed the upregulation of IL-6-JAK gene expression in HCMV-DB-infected HMECs compared to uninfected HMECs which has been reported to play a role in the appearance of tumorspheres in cultures of hepatocytes infected with HCMV^10^. In addition, the gene expression of alpha 6 integrin (ITGA6), a marker of stemcellness, is upregulated in HCMV-DB-infected HMECs. Finally in HCMV-DB-infected HMECs, the proto-oncogene tyrosine-protein kinase Src^87^, a gene involved in EMT, is upregulated compared to uninfected HMECs. Altogether our results indicate a potential trend towards the appearance of cancer stem cells (CSCs) in HMECs infected with HCMV-DB. CSCs are required for sustained tumor growth, invasiveness and metastasis formation^88^. The expansion of CSCs can be measured by the formation of tumorspheres. In agreement with the activation of IL-6-JAK pathway, upregulation of alpha 6 integrin and src gene expression, we observed tumorsphere formation in cultures of HCMV-DB-infected HMECs.

In conclusion, we define here the genomic profile of a newly isolated clinical HCMV strain, namely HCMV-DB, and its close phylogenetic links with other HCMV strains, especially the JP, Toledo and Merlin strains. Further, we confirmed that the HCMV-DB strain infects HMECs which display a triple negative phenotype. Our data indicate that the transcriptome of HCMV-DB-infected HMECs presents traits of a pro-oncogenic cellular environment with upregulated expression of numerous oncogenes, enhanced activation of pro-survival genes, and upregulated markers of cell proliferation, stemcellness and EMT that was confirmed by enhanced tumorsphere formation *in vitro*.

## Materials and Methods

### Reagents

Anti-US28 and anti β-actin antibodies were respectively acquired from Santa Cruz Biotechnology (Santa Cruz, CA) and Sigma-Aldrich (St. Louis, MO).

### Cell cultures

HMECs were purchased from Life Technologies (Carlsbad, CA, USA). MDA-MB231 and MCF-7 cells were provided by Institut Hiscia (Arlesheim, Switzerland). Cell culture and cell viability assay was performed as previously described^8^. Cultures were free of mycoplasma.

### Infection of HMECs with HCMV

The clinical isolate HCMV-DB was previously isolated in our laboratory^8^. Cell-free virus stocks of HCMV-DB were grown in macrophages, as described previously^8^. Human fibroblasts (MRC5) were cultured as previously described^6,8^. Cells infection by HCMV, heat-inactivated HCMV, UV-treated HCMV was performed as previously described^29^. Virus titers were determined by plaque-forming assay in MRC5 as described previously^8^. Viral stocks purity and viral replication were assessed as previously described^29^. Quantification of viral titer was performed by qPCR on cell culture supernatants as previously described^8^.

### Western blotting

pUS28 and β-actin expression was examined by western blotting as described previously^8^, using infected or uninfected HMECs cell lysates.

### RT^2^ Profiler^TM^ PCR Arrays

Total RNA was extracted from uninfected HMECs and HMECs infected with HCMV-DB at MOI of either 1 or 10 at day 1 post infection using TRIzol reagent (Life Technologies, Grand Island, NY), and the first strand cDNA synthesis was achieved using the RT^2^ First Strand Kit (SABiosciences, Valencia, CA) following the manufacturer’s instructions. Three RT^2^ Profiler^TM^ PCR Arrays for human breast cancer (PAHS-131ZA), oncogenes/tumor suppressor genes (PAHS-502Z) and human epigenetic chromatin modification enzymes (PAHS-085A) (all three from SABiosciences) were applied on the Strategene Mx3005P real-time PCR system (Agilent Technology, Santa Clara, CA) as per manufacturer’s instructions. Housekeeping genes, contamination control, reverse transcription control and positive controls were included in each PCR according to manufacturer’s instructions. Data analyses were performed using the web based analysis software (http://pcrdataanalysis.sabiosciencescom/pcr/arrayanalysis.php).

### Tumorsphere assay

Tumorsphere formation by uninfected HMECs or by HMECs infected using HCMV-DB or UV-inactivated HCMV-DB, was assayed as described previously^10^. MCF-7 and MDA-MB231 were used as positive controls. The number of tumorspheres larger than 60 microns was counted.

### Genomic analysis of HCMV-DB

The analysis of HCMV-DB genome and its comparison to other HCMV strains: AD-169 and Towne (laboratory adapted strains) and Merlin, Toledo, TR, PH, VR1814, Davis, JP, and TB40/E (clinical strains with low passages in culture) was done using the NCBI nucleotide blast tool (https://blast.ncbi.nlm.nih.gov/BlastAlign.cgi).

### Phylogenetic analysis

Phylogenetic proximity was calculated among various HCMV strains (described in Table 1) for UL55, UL75, UL115, UL128, UL130, and UL131 genes. The phylogenetic analysis was performed as previously reported^89^.

### Statistical analysis

Numerical values are shown as the means and SD of independent experiments. Mann Whitney U test was performed for statistical significance and differences were considered significant at a value of *P* < 0.05. The plots were prepared using Microsoft Excel.

### Availability of data and materials

The datasets used and/or analyzed during the present study are available from the corresponding author on reasonable request.

## Electronic supplementary material

Dataset1
