# Supplementary material for: The transcriptome of human mammary epithelial cells infected with the HCMV-DB strain displays oncogenic traits
Source: Sci Rep. 2018 Aug 22;8:12574. doi: 10.1038/s41598-018-30109-1 (PMC6105607; doi:10.1038/s41598-018-30109-1)
Supplement: Supplementary file 1 — Dataset1 [file 41598_2018_30109_MOESM1_ESM.pdf]

# The transcriptome of human mammary epithelial cells infected with the HCMV-DB strain displays oncogenic traits.

Fatima Al Moussawi[1,2,+], Amit Kumar[1,+], Sébastien Pasquereau[1], Manoj K. Tripathy[1], Walid Karam[2], Mona Diab-Assaf [2] & Georges Herbein[1,3,*].

**Suppl. Fig. 1** Upregulation of the tumor suppressor gene hypermethylated in cancer 1 (HIC1) and forkhead box D3 (FOXD3) in HMECs infected with HCMV-DB.

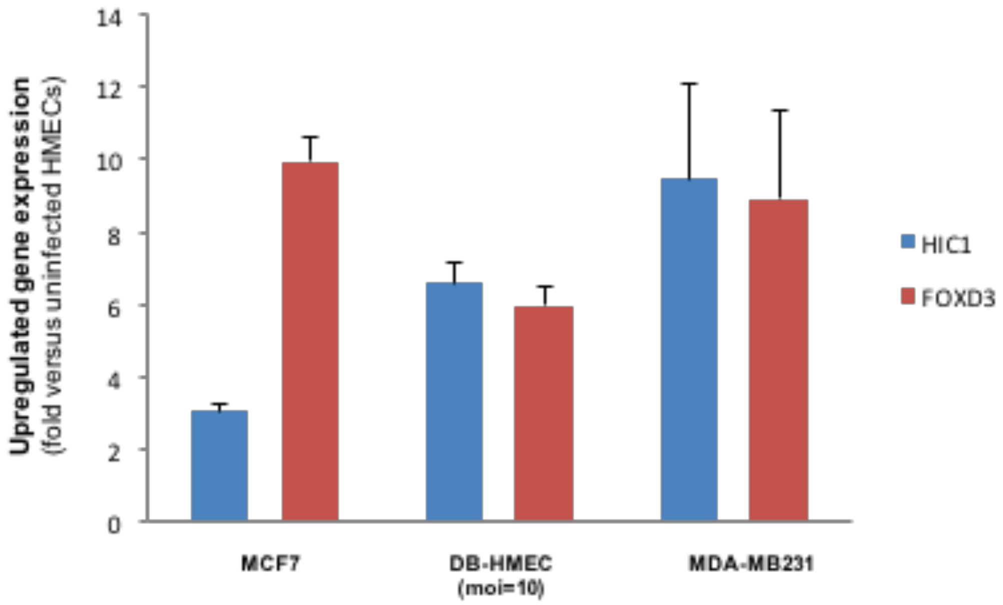

**Suppl. Table 1.** Degree of variability between the ORF of the 11 HCMV strains studied.

| Conserved genes | | Variable genes | | Highly Variabl | |
|---|---|---|---|---|---|
| IRS1 | | RL10 | | RL1 | |
| RL11 | | RL8A | | RL12 | |
| RNA1.2 | | S30 | | RL13 | |
| RNA2.7 | | UL10 | | RL5A | |
| RNA4.9 | | UL11 | | RL6 | |
| RNA5.0 | | UL112 | | RL9 | |
| TRS1 | | UL115 | | RL9A | |
| U13 | | UL121 | | UL1 | |
| UL100 | | UL124 | | UL105 | |
| UL102 | | UL130 | | UL119 | |
| UL103 | | UL132 | | UL120 | |
| UL104 | | UL135 | | UL128 | |
| UL111A | | UL138 | | UL133 | |
| UL114 | | UL139 | | UL139 | |
| UL116 | | UL14 | | UL144 | |
| UL117 | | UL140 | | UL146 | |
| UL122 | | UL142 | | UL147 | |
| UL123 | | UL148 | | UL147A | |
| UL131A | | UL148B | | UL148A | |
| UL136 | | UL150A | | UL148D | |
| UL141 | | UL17 | | UL150 | |
| UL145 | | UL19 | | UL2 | |
| UL148C | | UL20 | | UL33 | |
| UL15A | | UL22A | | UL4 | |
| UL16 | | UL27 | | UL40 | |
| UL18 | | UL30 | | UL47 | |
| UL21A | | UL40 | | UL6 | |
| UL23 | | UL41A | | UL61 | |
| UL24 | | UL45 | | UL73 | |
| UL25 | | UL49 | | UL74A | |
| UL26 | | UL55 | | UL74A | |
| UL29 | | UL7 | | UL75 | |
| UL31 | | UL76 | | UL9 | |
| UL32 | | UL78 | | US1 | |
| UL34 | | UL8 | | US10 | |
| UL35 | | UL80 | | US27 | |
| UL36 | | UL87 | | US33A | |
| UL37 | | UL93 | | US7 | |
| UL38 | | UL99 | | | |
| UL42 | | US23 | | | |
| UL43 | | US26 | | | |
| UL44 | | US28 | | | |
| UL46 | | US33A | | | |
| UL47 | | US6 | | | |
| UL48 | | US9 | | | |
| UL48A | | | | | |
| UL5 | | | | | |
| UL50 | | | | | |
| UL51 | | | | | |
| UL52 | | | | | |
| UL53 | | | | | |
| UL54 | | | | | |
| UL56 | | | | | |
| UL57 | | | | | |
| UL69 | | | | | |
| UL70 | | | | | |
| UL71 | | | | | |
| UL72 | | | | | |
| UL77 | | | | | |
| UL79 | | | | | |
| UL80.5 | | | | | |
| UL82 | | | | | |
| UL83 | | | | | |
| UL84 | | | | | |
| UL85 | | | | | |
| UL86 | | | | | |
| UL88 | | | | | |
| UL89 | | | | | |
| UL91 | | | | | |
| UL92 | | | | | |
| UL94 | | | | | |
| UL95 | | | | | |
| UL96 | | | | | |
| UL97 | | | | | |
| UL98 | | | | | |
| US11 | | | | | |
| US12 | | | | | |
| US13 | | | | | |
| US14 | | | | | |
| US15 | | | | | |
| US16 | | | | | |
| US17 | | | | | |
| US18 | | | | | |
| US19 | | | | | |
| US20 | | | | | |
| US21 | | | | | |
| US21 | | | | | |
| US22 | | | | | |
| US24 | | | | | |
| US29 | | | | | |
| US3 | | | | | |
| US31 | | | | | |
| US32 | | | | | |
| US34 | | | | | |
| US8 | | | | | |

**Suppl. Table 2.** Cellular gene expression in HMECs infected with HCMV-DB compared to unifected HMECs using the human breast cancer genes RT$^2$ profiler PCR assays (PAHS-131ZA).

| | | Fold Up- or Down- Regulation/ HMEC UI | | | |
|---|---|---|---|---|---|
| **Symbol** | **Description** | **MCF-7** | **HMEC DB 1MOI** | **HMEC DB 10 MOI** | **MDAMB-231** |
| CCNA1 | Cyclin A1 | -1.68 | 3.62 | 6.73 | 7.03 |
| CCNA2 | Cyclin A2 | 5.44 | 3.83 | 10.49 | 16.27 |
| CCNE1 | Cyclin E1 | -2.11 | 4.05 | 8.52 | 3.08 |
| CDH1 | Cadherin 1. type 1. E-cadherin (epithelial) | ND | 6.33 | 7.54 | ND |
| CLDN7 | Claudin 7 | ND | 9.43 | 26.93 | ND |
| CTNNB1 | Catenin (cadherin-associated protein). beta 1. 88kDa | ND | 2.94 | 6.97 | ND |
| EGF | Epidermal growth factor | ND | 1.28 | 6.08 | ND |
| ESR1 | Estrogen receptor 1 | 875.39 | 2.34 | 24.26 | 5.40 |
| ESR2 | Estrogen receptor 2 (ER beta) | 16.15 | -2.46 | 47.54 | 41.18 |
| GATA3 | GATA binding protein 3 | 154.75 | 4.62 | 5.21 | 1.72 |
| GLI1 | Glioma-associated oncogene | ND | -3,71 | ND | ND |
| HER2 | Human epidermal growth factor receptor 2 | 5.45 | 4.30 | 32.84 | 10.06 |
| IGF1R | Insulin Like Growth Factor 1 Receptor | ND | -7,32 | ND | ND |
| ITGA6 | Integrin Subunit Alpha 6 | ND | 2.63 | 11.48 | ND |
| KRT18 | Keratin 18 | 59.46 | 0.01 | 5.86 | 5.75 |
| KRT19 | Keratin 19 | 34249.59 | 2.21 | 8.88 | 4717.98 |
| MAPK3 | Mitogen-Activated Protein Kinase 3 | ND | -10,36 | ND | ND |
| MAPK8 | Mitogen-Activated Protein Kinase 8 | ND | -5,59 | ND | ND |
| MKI67 | Antigen identified by monoclonal antibody Ki-67 | 15.93 | 4.10 | 10.86 | 14.46 |
| PGR | Progesterone receptor | 1482.48 | 0.09 | 57.32 | 8.02 |
| SERPINE1 | Serpin Family E Member 1 | ND | 2.41 | 26.37 | ND |
| S100A4 | S100 Calcium Binding Protein A4 | ND | 2.52 | 1.41 | ND |
| TFF1 | Trefoil factor 1 | 8294934.92 | 3.67 | 150.22 | 2126.04 |
| THBS1 | Thrombospondin 1 | ND | 3.15 | 7.07 | ND |
| TOPO2A | Topoisomerase (DNA) II alpha 170kDa | 16.04 | 6.09 | 36.02 | 22.85 |

**Suppl. Table 3.** Cellular gene expression in HMECs infected with HCMV-DB compared to unifected HMECs using the oncogenes/tumor suppressor genes RT$^2$ profiler PCR assays (PAHS-502Z).

| | | Fold Up- or Down- Regulation/ HMEC UI | | | |
|---|---|---|---|---|---|
| **Symbol** | **Description** | **MCF-7** | **HMEC DB 1MOI** | **HMEC DB 10 MOI** | **MDAMB-231** |
| ABL1 | C-abl oncogene 1. receptor tyrosine kinase | 4.17 | 4.73 | 4.24 | 2.07 |
| AKT1 | V-akt murine thymoma viral oncogene homolog 1 | 12.13 | 4.00 | 1.43 | 2.05 |
| ATM | Ataxia telangiectasia mutated | 2.59 | 4.03 | 5.37 | 5.19 |
| BCL-2 | B-cell CLL/lymphoma 2 | 6.04 | 2.51 | 8.52 | 65.52 |
| CASP8 | Caspase 8. apoptosis-related cysteine peptidase | 4.29 | -3.36 | 4.87 | 1.42 |
| CCND1 | Cyclin D1 | 4.12 | 5.76 | 9.01 | 15.18 |
| CDK4 | Cyclin-dependent kinase-4 | 21.87 | 3.73 | 2.98 | 3.04 |
| CDKN1A | Cyclin-dependent kinase inhibitor 1A (p21. Cip1) | 3.33 | 3.12 | 4.39 | -6.53 |
| CDKN2A | Cyclin-dependent kinase inhibitor 2A (melanoma. p16. inhibits CDK4) | -513.42 | 5.79 | 4.55 | -641.81 |
| CDKN2B | Cyclin-dependent kinase inhibitor 2B (p15. inhibits CDK4) | 4.15 | 3.21 | 5.37 | -1046.53 |
| CDKN3 | Cyclin-dependent kinase inhibitor 3 | 24.35 | 7.58 | 4.04 | 2.78 |
| CST6 | Cystatin-M | ND | -3.61 | ND | ND |
| CTSD | Cathepsin D | ND | -1.85 | ND | ND |
| ETS1 | V-ets erythroblastosis virus E26 oncogene homolog 1 (avian) | -730.50 | 8.95 | 5.96 | 3.37 |
| FHIT | Fragile histidine triad gene | 1.68 | 2.25 | 3.96 | 0.16 |
| FOS | V-fos FBJ murine osteosarcoma viral oncogene homolog | -1.56 | 14.04 | 4.94 | -4.96 |
| FOXD3 | Forkhead box D3 | 9.92 | ND | 5.96 | 8.91 |
| HIC1 | Hypermethylated in cancer 1 | 3.08 | ND | 6.61 | 9.48 |
| HRAS | V-Ha-ras Harvey rat sarcoma viral oncogene homolog | 2.35 | 4.23 | 2.74 | -2.08 |
| ID1 | Inhibitor of DNA binding 1, dominant negative helix-loop-helix protein | 191.84 | -5.24 | 3.76 | 2.30 |

| | | Fold Up- or Down- Regulation/ HMEC UI | | | |
|---|---|---|---|---|---|
| **Symbol** | **Description** | **MCF-7** | **HMEC DB 1MOI** | **HMEC DB 10 MOI** | **MDAMB-231** |
| IL6 | Interleukin 6 (interferon. beta 2) | -4.43 | -3.89 | 16.92 | 331.75 |
| IL6R | Interleukin 6 receptor | 2.93 | 3.65 | 9.19 | -6.31 |
| IL6ST | Interleukin 6 signal transducer (gp130. oncostatin M receptor) | 5.40 | 4.91 | 22.80 | 32.99 |
| JAK2 | Janus kinase 2 | 14.04 | 4.53 | 7.34 | 2.82 |
| JUN | Jun oncogene | 6.50 | 5.10 | 1.99 | 3.80 |
| KITLG | KIT ligand | 3.27 | 9.13 | 2.98 | 2.40 |
| KRAS | V-Ki-ras2 Kirsten rat sarcoma viral oncogene homolog | 5.39 | 8.12 | 2.17 | 1.68 |
| MCL1 | Myeloid cell leukemia sequence 1 (BCL2-related) | 5.43 | 4.50 | 2.86 | -1458.21 |
| MET | Met proto-oncogene (hepatocyte growth factor receptor) | -6.60 | 5.74 | 3.06 | 1.48 |
| MLH1 | MutL homolog 1. colon cancer. nonpolyposis type 2 (E. coli) | 9.01 | 3.98 | 3.72 | 3.10 |
| MMP9 | Matrix metallopeptidase 9 | ND | 7,91 | ND | ND |
| MOS | V-mos Moloney murine sarcoma viral oncogene homolog | ND | -2.60 | ND | ND |
| MYB | V-myb myeloblastosis viral oncogene homolog (avian) | 1060.90 | 2.19 | 2.78 | 13.31 |
| MYC | V-myc myelocytomatosis viral oncogene homolog (avian) | 1.59 | 9.86 | 3.72 | 1.45 |
| NFKB1 | Nuclear factor of kappa light polypeptide gene enhancer in B-cells 1 | 4.47 | 3.63 | 1.52 | 0.01 |
| NFKBIA | Nuclear factor of kappa light polypeptide gene enhancer in B-cells 1 | 6.11 | 2.81 | 1.53 | 1.28 |
| NRAS | Neuroblastoma RAS viral (v-ras) oncogene homolog | 35.53 | 8.52 | 1.96 | 3.67 |
| PIK3C2A | Phosphoinositide-3-kinase, class 2, alpha polypeptide | 5.32 | 7.07 | 2.32 | 1.88 |

| | | Fold Up- or Down- Regulation/ HMEC UI | | | |
|---|---|---|---|---|---|
| **Symbol** | **Description** | **MCF-7** | **HuMEC DB 1MOI** | **HuMEC DB 10 MOI** | **MDAMB-231** |
| PIK3CA | Phosphoinositide-3-kinase, catalytic, alpha polypeptide | 5.28 | 5.82 | 2.84 | 6.30 |
| PML | Promyelocytic leukemia | 2.45 | 10.13 | 1.78 | 2.84 |
| PRKCA | Protein kinase C. alpha | 8.52 | 6.24 | 2.29 | 35.88 |
| RAF1 | V-raf-1 murine leukemia viral oncogene homolog 1 | 13.56 | 5.32 | 2.34 | 2.29 |
| RARA | Retinoic acid receptor. alpha | 4.96 | 7.32 | -1.20 | 8.72 |
| RB1 | Retinoblastoma 1 | 3.72 | 4.16 | 1.52 | 6.75 |
| REL | V-rel reticuloendotheliosis viral oncogene homolog (avian) | 3.97 | 3.76 | -1.14 | 0.23 |
| RET | Ret proto-oncogene | 515.94 | 6.59 | 3.59 | 1.81 |
| ROS1 | C-ros oncogene 1 . receptor tyrosine kinase | -4.67 | 6.07 | 4.01 | -5.73 |
| RUNX1 | Runt-related transcription factor 1 | 3.37 | 3.92 | 1.37 | 2.94 |
| RUNX3 | Runt-related transcription factor 3 | -1.23 | 2.83 | 5.96 | 2.82 |
| SLIT2 | Slit homolog 2 (Drosophila) | -71.36 | -16.68 | -90.57 | -1304.20 |
| SMAD4 | SMAD family member 4 | 2.14 | 3.39 | 1.10 | 4.10 |
| STAT3 | Signal transducer and activator of transcription 3 (acute-phase response factor) | -1.30 | 1.27 | 1.50 | -0.03 |
| STK11 | Serine/threonine kinase 11 | 2.45 | 0.79 | 0.65 | -1.61 |
| TGFB1 | Transforming growth factor. beta 1 | -2.55 | 2.55 | -1.23 | 1.95 |
| TP53 | Tumor protein p53 | 4.00 | 5.28 | -1.20 | 7.49 |
| TP73 | Tumor protein p73 | 14.63 | 1.87 | 3.42 | 33.71 |
| TSC1 | Tuberous sclerosis 1 | 4.00 | 2.10 | 1.16 | 3.00 |
| VHL | Von Hippel-Lindau tumor suppressor | 9.52 | 3.58 | 1.07 | 3.30 |

**Suppl. Table 4.** Cellular gene expression in HMECs infected with HCMV-DB compared to unifected HMECs using the Human Epigenetic Chromatin Modification Enzymes RT$^2$ Profiler PCR Array (PAHS-085A).

| Symbol | Description | Fold Up- or Down- Regulation HMEC DB / HMEC UI |
|---|---|---|
| ASH1L | ASH1 Like Histone Lysine Methyltransferas | -155.00 |
| ATF2 | Activating Transcription Factor 2 | -9.10 |
| AURKA | Aurora Kinase A | -8.20 |
| AURKB | Aurora Kinase B | -3.96 |
| AURKC | Aurora Kinase C | -2.15 |
| CIITA | Class II Major Histocompatibility Complex Transactivator | -3.91 |
| CSRP2BP | CSRP2 binding protein | -6.26 |
| DNMT3A | DNA Methyltransferase 3 Alpha | -2.94 |
| DNMT3B | DNA Methyltransferase 3 Beta | -1.42 |
| HDAC10 | Histone Deacetylase 10 | -2.05 |
| HDAC2 | Histone Deacetylase 2 | -19.11 |
| HDAC3 | Histone Deacetylase 3 | 7.43 |
| HDAC4 | Histone Deacetylase 4 | -19.64 |
| HDAC5 | Histone Deacetylase 5 | -3.57 |
| HDAC6 | Histone Deacetylase 6 | -2.47 |
| HDAC9 | Histone Deacetylase 9 | -2.03 |
| KAT2A | Lysine acetyltransferase 2A | -12.26 |
| KAT6B | Lysine acetyltransferase 6B | -17.34 |
| KDM1A | Lysine demethylase 1A | -16.75 |
| KDM5C | Lysine demethylase 5C | -3.40 |
| KDM4C | Lysine demethylase 4C | -15.23 |
| KDM6B | Lysine demethylase 6B | -12.43 |
| MLL3 (KMT2C) | Lysine methyltransferase 2C | -3.54 |
| MLL5 (KMT2E) | Lysine methyltransferase 2E | -4.98 |
| MYSM1 | myb-like. SWIRM and MPN domains 1 | -2.69 |
| NCOA1 | Nuclear receptor coactivator 1 | -3.52 |
| NEK6 | NIMA related kinase 6 | -11.76 |
| NSD1 | Nuclear receptor binding SET domain protein 1 | -2.82 |

| Symbol | Description | Fold Up- or Down- Regulation HMEC DB / HMEC UI |
|---|---|---|
| | WHSC1 | Wolf-Hirschhorn syndrome candidate 1 |
| PRMT1 | Protein arginine methyltransferase 1 | -2.63 |
| PRMT2 | Protein arginine methyltransferase 2 | -2.63 |
| PRMT3 | Protein arginine methyltransferase 3 | 2.68 |
| PRMT5 | Protein arginine methyltransferase 5 | 2.76 |
| PRMT6 | Protein arginine methyltransferase 6 | 2.84 |
| PRMT8 | Protein arginine methyltransferase 8 | -22.41 |
| RNF2 | Ring finger protein 2 | -551.09 |
| RNF20 | Ring finger protein 20 | -84.81 |
| RPS6KA3 | Ribosomal protein S6 kinase A3 | -554.92 |
| SETD1A | SET domain containing 1A | -7.39 |
| SETDB1 | SET domain bifurcated 1 | -18.58 |
| SETDB2 | SET domain bifurcated 2 | -76.96 |
| SETD2 | SET domain containing 2 | -3.85 |
| SETD3 | SET domain containing 3 | -3.54 |
| SETD4 | SET domain containing 4 | 2.22 |
| SETD5 | SET domain containing 5 | -8.73 |
| SETD6 | SET domain containing 6 | -7.98 |
| SETD7 | SET domain containing 7 | -51.84 |
| SETD8 | SET domain containing 8 | -4056.84 |
| SMYD3 | SET and MYND domain containing 3 | -2.45 |
| SUV39H1 | Suppressor of variegation 3-9 homolog 1 | -5.72 |
| SUV420H1 | Suppressor of variegation 4-20 homolog 1 | -26.10 |
| UBE2A | Ubiquitin conjugating enzyme E2 A | -11.92 |
| UBE2B | Ubiquitin conjugating enzyme E2 B | -3.99 |
| USP21 | Ubiquitin specific peptidase 21 | -94.10 |
| USP22 | Ubiquitin specific peptidase 22 | -31.69 |
| WHSC1 | Wolf-Hirschhorn syndrome candidate 1 | -32.13 |

**Suppl. Table 5.** Cellular gene families classification of the Human Epigenetic Chromatin Modification Enzymes RT$^2$ Profiler PCR Array (PAHS-085A).

| Family | Symbol | Description |
|---|---|---|
| Histone acetyltransferase genes | ATF2 | Activating Transcription Factor 2 |
| | CIITA | Class II Major Histocompatibility Complex Transactivator |
| | CSRP2BP | CSRP2 binding protein |
| | KAT2A | Lysine acetyltransferase 2A |
| | KAT6B | Lysine acetyltransferase 6B |
| | NCOA1 | Nuclear receptor coactivator 1 |
| Histone kinase genes | AURKA | Aurora Kinase A |
| | AURKB | Aurora Kinase B |
| | AURKC | Aurora Kinase C |
| | NEK6 | NIMA related kinase 6 |
| | RPS6KA3 | Ribosomal protein S6 kinase A3 |
| Genes involved in histone ubiquitination | MYSM1 | Myb Like SWIRM and MPN domains 1 |
| | RNF2 | Ring finger protein 2 |
| | RNF20 | Ring finger protein 20 |
| | UBE2A | Ubiquitin conjugating enzyme E2 A |
| | UBE2B | Ubiquitin conjugating enzyme E2 B |
| | USP21 | Ubiquitin specific peptidase 21 |
| | USP22 | Ubiquitin specific peptidase 22 |
| DNA/histone demethylase genes | KDM1A | Lysine demethylase 1A |
| | KDM5C | Lysine demethylase 5C |
| | KDM4C | Lysine demethylase 4C |
| | KDM6B | Lysine demethylase 6B |

| **Family** | **Symbol** | **Description** |
|---|---|---|
| Histone deacetylase genes | HDAC10 | Histone Deacetylase 10 |
| | HDAC2 | Histone Deacetylase 2 |
| | HDAC3 | Histone Deacetylase 3 |
| | HDAC4 | Histone Deacetylase 4 |
| | HDAC5 | Histone Deacetylase 5 |
| | HDAC6 | Histone Deacetylase 6 |
| | HDAC9 | Histone Deacetylase 9 |
